# Structure and Sequence Aligned Code Summarization with Prefix and Suffix Balanced Strategy

**DOI:** 10.3390/e25040570

**Published:** 2023-03-26

**Authors:** Jianhui Zeng, Zhiheng Qu, Bo Cai

**Affiliations:** Key Laboratory of Aerospace Information Security and Trusted Computing, Ministry of Education, School of Cyber Science and Engineering, Wuhan University, Wuhan 430072, China

**Keywords:** source code summarization, deep learning, program comprehension

## Abstract

Source code summarization focuses on generating qualified natural language descriptions of a code snippet (e.g., functionality, usage and version). In an actual development environment, descriptions of the code are missing or not consistent with the code due to human factors, which makes it difficult for developers to comprehend and conduct subsequent maintenance. Some existing methods generate summaries from the sequence information of code without considering the structural information. Recently, researchers have adopted the Graph Neural Networks (GNNs) to capture the structural information with modified Abstract Syntax Trees (ASTs) to comprehensively represent a source code, but the alignment method of the two information encoder is hard to decide. In this paper, we propose a source code summarization model named SSCS, a unified transformer-based encoder–decoder architecture, for capturing structural and sequence information. SSCS is designed upon a structure-induced transformer with three main novel improvements. SSCS captures the structural information in a multi-scale aspect with an adapted fusion strategy and adopts a hierarchical encoding strategy to capture the textual information from the perspective of the document. Moreover, SSCS utilizes a bidirectional decoder which generates a summary from opposite direction to balance the generation performance between prefix and suffix. We conduct experiments on two public Java and Python datasets to evaluate our method and the result show that SSCS outperforms the state-of-art code summarization methods.

## 1. Introduction

Source code summarization is a popular research task in the code comprehension field which aims to generate natural language descriptions of code for developers to rapidly comprehend the functionality or usage. With the increasing volume of software code, nearly 90% of the development cost is spent on software maintenance, (e.g., version iteration, program comprehension and bug fixing) [1]. High-quality code summaries undoubtedly can effectively reduce the cost on program comprehension. Nowadays, in a practical developing environment, most of the code summaries are likely to be missing or lacking, or the summaries do not match the code due to a series of human mistakes or the large volume of code. Developers’ effort in writing qualified summaries determines whether code can be effectively comprehended by other developers. Recently, researchers are devoted to source code summarization tasks to generate high-quality code summaries automatically instead of hand-writing, and it is still challenging.

Early studies on code summarization tasks mainly generated a summary from templates [2] or retrieved a summary from a similar code snippet based on information retrieval (IR) techniques [3]. Whether the template-based approaches or the IR-based approaches, the quality of the summaries is far from satisfactory.

Recently, due to the remarkable achievement in neural machine translation tasks utilizing deep learning techniques, studies on automatic code summarization based on a sequence-to-sequence architecture that contains an encoder for code representation and a decoder for the summary generation attracts researchers devoted in this area. Researchers [4,5,6] only consider the code as plain text data, which is called sequential information (e.g., token sequence, API sequence) and generate summaries based on it with an RNN (Recurrent Neural Network) or CNN (Convolutional Neural Network) sequence-to-sequence model. Further, transformer [7] architecture shows great potential in the Natural Language Processing (NLP) area providing a breakthrough for all language-related tasks. As a result, works such as [8,9] on using transformers to generate summaries, achieved better results than the RNN-based or CNN-based methods. Ahmad et al. [8] first proposed a transformer-based method on the code summarization task, which achieved excellent performance and leads the code summarization area into the transformer-based model stage. Because of the popularization and performance of transformers, almost all recent works [9,10,11,12] are conducted based on the transformer architecture and achieve high scores in each evaluation metric. However, only considering sequence information without considering the structure of code leads to a incomplete representation of code. Thus, researchers utilized the structural information of code (e.g., Abstract Syntax Tree (AST), Control Flow Graph (CFG), Data Flow Graph (DFG), Program Dependence Graph (PDG)) to represent code more comprehensively. Specifically, a part of code summarization research [13,14,15] used ASTs as the input to generate code summaries. Shido et al. [16] proposed an LSTM-based (Long Short-term Memory Network) tree structure model to directly capture AST features that enable a traditional NLP model to apply to tree-structural data. Alon et al. [15] extracted multiple tree paths from root node to terminal node based on ASTs as the code representations as structural information for summary generation. Currently, Graph Neural Networks (GNNs) achieve great performance on graph data (e.g., protein prediction, knowledge graph), so researchers [17,18] consider constructing AST into a graph by adding additional edge relations and utilizing GNNs to capture structural information achieving great results.

Generally, to comprehensively represent source code and achieve great generation performance, it is undoubtedly that we must consider both structure and sequence information, thus leading to the two motivations for our work. The first motivation of this paper is to capture the sequence information in a hierarchical view while almost all the works consider code sequence as plain text, despite the fact that codes are a kind of document data containing hierarchical structure. Code is not only a composition of tokens but also a composition of statement sentences, which also means that code contains document property. Thus, to better capture the sequence information, we propose a hierarchical sequence encoder adopting a hierarchical encoding strategy from token-level to sentence-level. For the second motivation, code ASTs or graphs often contain large scale nodes and edges for which the GNNs do not have the parallel computation ability unless we combine batches of code ASTs or graphs into a larger graph, leading to low computational efficiency. Thus, we utilize the high parallel computation ability of transformers to encode a batch of code graphs with a special mask technique. Furthermore, for natural language generation (NLG) tasks, the decoder is an auto-regressive module which generates sentences from left to right, suffering from an exposure bias issue that error will be accumulated and passed to the following step. In conclusion, this leads to an unbalanced performance between prefix and suffix. The prefix denotes the first *m* tokens in a generated sentence and the suffix denotes the last *n* tokens. Thus, we adopt a bidirectional decoding strategy to balance the performance between prefix and suffix. We will detail the above designs in Section 3.

**Contributions:**

- We propose a structure and sequence-aligned code summarization method called SSCS, which can not only capture both types of information in a way superior to the previous works but also balances the generating performance between prefix and suffix.
- We design a transformer-based AST encoder to explicitly encode the structural information in a multi-perspective format and aggregate it with an adapted fusion strategy.
- A hierarchical sequence encoder is proposed to capture the document property of code, which enables the model to understand sequence information from fine grain (token-level) to coarse grain (sentence-level).
- We conduct experiments on two public datasets on Java and Python to evaluate the effectiveness of our proposed SSCS. Furthermore, the results show that our approach outperforms exiting state-of-the-art code summarization methods on BLEU, METEOR and ROUGE metrics.

## 2. Related Work

In early periods of code summarization task, researchers generated natural language descriptions of code based on the Software Word Usage Model (SWUM) by analyzing the signatures of Java methods [2]. Subsequently, Information Retrieval (IR) techniques emerged and were widely used in code summarization tasks. Haiduc et al. [3] proposed IR-based techniques for automatic code summarization, which searches similar code snippets or keywords in the code database and extracts the summary from similar ones as the summary of the original code. These methods generate code summaries with low flexibility. Recently, researchers utilized deep-learning techniques to represent code and adopt the encoder–decoder framework to generate a more flexible summary. To our best knowledge, recent methods can be broadly divided into three categories: sequence-only methods, structure-aligned methods and pre-trained methods.

As the encoder–decoder framework achieves great success in Neural Machine Translation (NMT), which is able to generate sentences of arbitrary length, researchers consider code summarization as a translation task that translates program language (PL) into natural language (NL). Thus, studies generate summaries with sequence information of the code (e.g., code plain text, API sequence). Iyer et al. [4] were the first to propose CODE-NN, an RNN-based encoder–decoder framework equipped with an attention mechanism, generating a summary from plain text. Allamanis et al. [5] utilized a Convolutional Attention Neural Network (CNN) to generate a function-name-level short summary for specific code. Hu et al. [6] designed a method which utilizes API sequences as complementary information for the code sequence, adding an extra API sequences encoder to represent the API sequence. Wei et al. [19], who regarded code summarization and code generation as complementary tasks, proposed a dual learning model to learn both code and summaries simultaneously, improving the performance of each task. The emergence of transformers [7] brought huge progress in the natural language generation (NLG) field, becoming the mainstream architecture for the subsequent works. Ahmad et al. [8] first proposed a transformer-based method to generate code summaries which add a copy mechanism and relative positional encoding strategy to strengthen the absolute position encoding strategy. Moreover, Wan et al. [1] utilized reinforcement learning strategy, adding an extract actor–critic module to conduct the generation performance. The above methods generated summaries with sequence information only and demonstrated the potential of deep-learning techniques in the code summarization field.

Only considering the source code as a sequence ignores the structural property of the code and leads to an incomplete representation of the code, thus the performance of the code summarization task encounters a bottleneck. To overcome the bottleneck caused by incomplete representation, researchers constructed a more comprehensive representation by adding structure information from ASTs. Hu et al. [13] proposed a transformation algorithm that transforms tree-structure data from AST into sequence data SBT (structure-based traversal) and adopted the RNN-based encoder–decoder framework to generate the summary. LeClair et al. [14] utilized both AST sequence and code sequence with two separate encoders to capture the different kinds of information. Liang and Zhu [20] proposed a tree-based recursive neural network to directly capture AST data instead of transforming the AST into a sequence. Shido et al. [16] proposed tree-LSTM to capture the structural information. Fernandes et al. [21] constructed sequence graphs of code and used Gated Graph Neural Networks (GGNN) [22] as the encoder that can capture the distance relationship in the sequence graphs. Alon et al. [15] extracted multiple paths from the AST, constructing a path-based sequence to represent the structure of code. LeClair et al. [23] proposed a graph-based neural network which uses an RNN-based network to capture the sequence information and GCN for the structural information. Wang et al. [24] generated code summary by aggregating source code sequence information, ASTs and a control-flow graph with reinforce learning strategy. In general, by integrating sequence information and structural information, code summarization has made great progress but there is still a barrier to satisfy the needs of industry.

Recently, due to great improvement provided by pre-trained models (e.g., BERT [25], T5 [26]), some researchers designed pre-trained models specifically for code representation. Feng et al. [27] proposed pre-trained model CodeBert which considers the representation of the data flow of code, achieving promising results in the code summarization task. Furthermore, Wang et al. [28] proposed CodeT5 based on T5 [26] considering the token type of code and utilizing a denoising sequence-to-sequence pre-training strategy. The above code pre-trained models generally understand deeper relationship within codes by feeding large enough data, and the results are pretty impressive.

## 3. Proposed Approach

### 3.1. Overview

**Figure 1:**
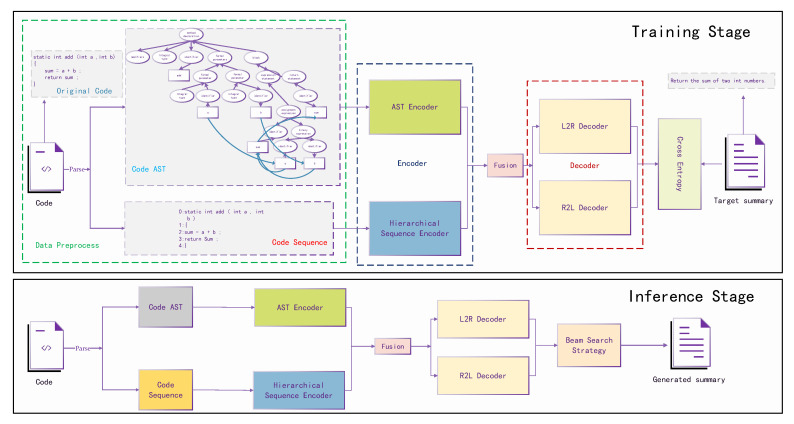
Overall process of SSCS. A model with two unique encoders for sequence information and structure information is followed by a bidirectional decoding module for left-to-right decoding and right-to-left decoding.

The overview of our proposed method(SSCS) is shown in Figure 1.

To detail our proposed method, we separate the whole process into two stages, training stage and inference stage. We can clearly see that SSCS contains three main components encoder, fusion module and decoder, which will be detailed in the following section.

As shown in Figure 1, code and ground-truth summary are both utilized in the training stage, while only code will be utilized in the inference stage. First the code will be processed into AST and lines of code sequence for the AST encoder and hierarchical sequence encoder, respectively, followed by a fusion module. Later the fusion output will be used by the decoder. The main difference between the training stage and inference stage is the process of the decoder. In the training stage, the ground-truth summary will be fed into the decoder and computes the loss with the decoder output for backward propagation, while the input is the generated token from the last step in the inference stage.

### 3.2. Data Preprocessing

The dataset we use for our work contains large <code, summary> pairs. We simply tokenize the summary into a list of tokens. Furthermore, to reduce out-of-vocabulary issues caused by a large scale of unique tokens, we tokenize any CamelCase or snake_case defined by developers. The process of acquiring sequence information for code is same as the summary but we maintain the document property of code. Thus, the code sequence information consists of multiple tokenized lines of code sub-sequence.

Knowing that SSCS is a structure and sequence aligned model, we also need to parse code into an AST to obtain structural information. In this paper, we evaluate our method on two public Java and Python datasets. We generate Abstract Syntax Trees (ASTs) with open-source tool tree-sitter (https://tree-sitter.github.io/ (accessed on 15 July 2022)) for Java code and ast (https://github.com/python/cpython/blob/master/Lib/ast.py (accessed on 26 July 2022)) module for Python code. Moreover, we also add extra data flow information to strengthen the structural information. As shown in Figure 1, the variable sum is computed from addition of *a* and *b*, and we can see in the Code AST that the leaf node sum has data relationships with variable *a* and variable *b*, respectively. Finally, we obtain the node sequence by pre-order traversal and its adjacency as the AST encoder inputs.

### 3.3. Token and Node Embedding

Before sending the node and token into the model, we need to vectorize the token and node. We first create a dictionary to calculate the total number of tokens and nodes separately, and use $num_{token}$ and $num_{node}$ to represent them. Thus, we are able to vectorize the token or node as one-hot vector, which is a $num_{token}$ or $num_{node}$ size vector consisting of a unique “1”, and “0”s for the rest of the positions. To accelerate the computation, we usually embed the discrete one-hot vector into dense vector of size dim. The most simple way is to multiply a matrix with a size of num × dim, and we are able to transform the vector space $R^{num}$ into dense vector space $R^{dim}$.

### 3.4. AST Encoder

**Figure 2:**
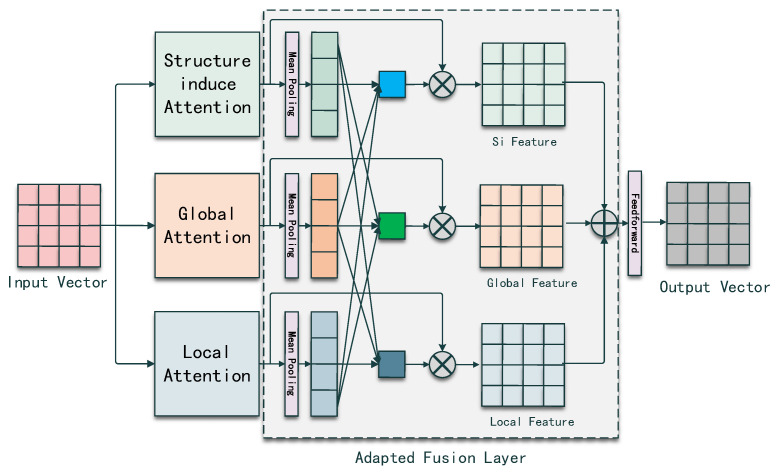
Overview of AST encoder. A encoder with three blocks, multi-view attention computation, adapted weight fusion and feed-forward network.

Wu et al. [9] proposed the SiT model which used transformer architecture to directly capture the structural information instead of using Graph Neural Networks that inspired our work. Inspired by SiT, we designed a transformer-based AST encoder capturing the structural information in multi-view. From the last section, we know that two inputs will be imported into the AST encoder, so we first define each input. Given an AST with *L* nodes $N=\{n_{1},n_{2},n_{3},\ldots ,n_{l}\}$, where $n_{j}\in R^{dim}$ denotes each node vector and dim denotes the dimension of node vector in vector space *R*. *A* denotes the adjacency matrix in the shape of *L*X*L*, the computation process of the AST encoder can be split into three blocks: multi-view attention computation, adapted weight fusion and feed-forward network. Figure 2 shows the overview of an AST encoder.

**Global Self-Attention** The computation of the global self-attention is based on the vanilla self-attention in transformers [7]. We treat the AST of the source code as an undirected complete graph, which means a node ni can learn the relation from the whole tree without any blocking. Therefore, we are able to capture the global AST representation. The global self-attention mechanism is denoted as follows:

(1) $$\begin{array}{c} SAN\left(N\right)=softmax\left(\frac{QK^{T}}{\sqrt{d_{k}}}\right)V \end{array}$$

(2) $$\begin{array}{c} Q,K,V=NW^{Q},NW^{K},NW^{V} \end{array}$$

where $N=\{n_{1},n_{2},\ldots ,n_{l}\}$ denotes the input sequence of nodes, *l* denotes the node sequence length and $d_{k}$ is the dimension of *K*. $W^{Q}$, $W^{K}$ and $W^{V}$, $W^{Q}$, $W^{K}$, $W^{V}\in R^{dim\times dim}$ are three learnable matrices using as projection to transform the vector space into a different vector space. $NW^{Q},NW^{K}$ and $NW^{V}$ represent matrix multiply operation (matrix N dot matrix W).

**Structure-induced Self-Attention** We follow the previous work by Wu et al. [9] to represent the structure information using transformer architecture equipped with a special attention mechanism. The structure-induced self-attention network (Si-SAN) is able to capture the structural information instead of using Graph Neural Networks (GNNs). The computation of the Si-San is to multiply the adjacency matrix by key-query pairs:

(3) $$\begin{array}{c} SiSAN\left(N\right)=softmax\left(\frac{A\cdot QK^{T}}{\sqrt{d_{k}}}\right)V \end{array}$$

(4) $$\begin{array}{c} A_{i,j}=\left\{\begin{array}{c} 1,\;if\;edge(i,j)\\ -inf,\;else \end{array}\right. \end{array}$$

where *A* denotes the adjacency matrix of the code. edge(i,j) denotes there is an edge between $n_{i}$ and $n_{j}$. The attention score between $n_{i}$ and $n_{j}$ will be dropped out when $a_{i,j}=-inf$ in *A*.

**Local Self-Attention** To further capture the structural information, we also adopt a local attention network to capture the local information. By adding a window mask, we can initialize a window which can slide through the whole tree to learn the local relation.

(5) $$\begin{array}{c} LSAN\left(N\right)=softmax\left(\frac{M_{win}\cdot QK^{T}}{\sqrt{d_{k}}}\right)V \end{array}$$

where $M_{win}$ denotes the window matrix for constraining the computation of node pairs in window distance.

**Adaptive Weight Fusion Layer** In SiT [9], the process of the encoder module is a global self-attention network followed by a structure-induced self-attention network, where the global information will be diluted by the Si-SAN. Thus, we adopt a superior fusion strategy for the different views of information by using an adaptive weight fusion layer, which is shown in Figure 2.

Given the outputs *G*, *S*, *L* from the SAN, SiSAN and LSAN, we first use a Mean Pooling Module to condense *G*, *S*, $L\in R^{dim\times dim}$ into $G^{\prime }$, $S^{\prime }$, $L^{\prime }\in R^{1\times dim}$.

(6) $$\begin{array}{c} G^{\prime },S^{\prime },L^{\prime }=MeanPooling\left(G,S,L\right) \end{array}$$

For vector $G^{\prime }$, $G^{\prime }$ obtains the relation weights by summing up the vectors after matrix multiplication with the rest of the two dense vectors and $S^{\prime }$ and $L^{\prime }$ repeat the same process. To simplify the computation, we join $G^{\prime }$, $S^{\prime }$ and $L^{\prime }$ and compute the dot value between the joint matrix and its transpose matrix. We sum up the relation weights and normalize as the adaptive weight for $G^{\prime }$. $S^{\prime }$ and $L^{\prime }$ repeat the same process to obtain the adaptive weight for themselves. The computation process is shown below.

(7) $$\begin{array}{c} s_{G},s_{S},s_{L}=Sum\left(\left[G^{\prime }:S^{\prime }:L^{\prime }\right]\times {\left[G^{\prime }:S^{\prime }:L^{\prime }\right]}^{T}\right) \end{array}$$

(8) $$\begin{array}{c} \alpha ,\beta ,\gamma =\sigma (s_{G}W_{G},s_{S}W_{S},s_{L}W_{L}) \end{array}$$

where $W_{G},W_{S},W_{L}\in R^{1\times 1}$,α,β,γ$\in R^{1\times 1}$ are the adaptive for *G*, *S* and *L*. σ refers to the $sigmoid\left(z\right)=\frac{1}{1+e^{-z}}$ activation function for normalizing the weight. “:” denotes the joint operation.

The final AST encoder output is the weighted sum of G,S and *L* followed by a Feed-Forward network.

(9) $$\begin{array}{c} Output_{ast}=FFN\left(\alpha \cdot G+\beta \cdot S+\gamma \cdot L\right) \end{array}$$

(10) $$\begin{array}{c} FFN\left(x\right)=ReLU\left(xW_{1}+b_{1}\right)W_{2}+b_{2} \end{array}$$

where *x* denotes the output from the adaptive weight fusion layer, $W_{1},W_{2}$ are two learnable matrices. For activation function ReLU, ReLU(x)=max(0,x).

**Figure 3:**
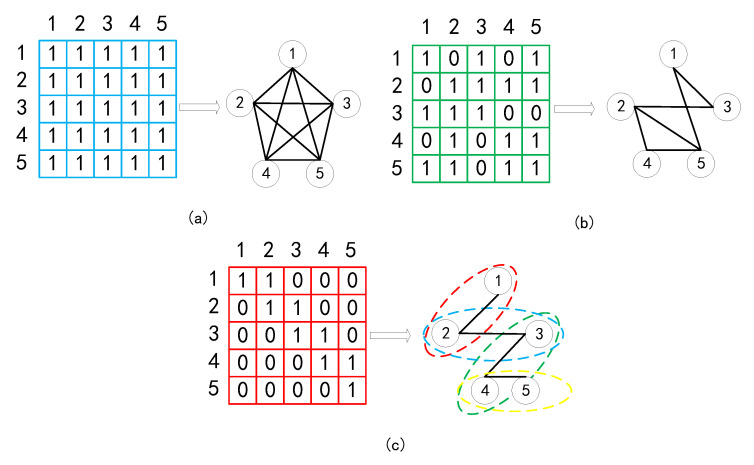
Three kinds of mask for capturing multi-view information. (**a**) Global mask for capturing global information. (**b**) Structure-induced mask for capturing structural information. (**c**) Window mask for capturing local information.

During attention computation stage, the $QK^{T}$ operation creates a square matrix, so we can utilize mask strategy to control the reception field. To better understand why the mask matrices are able to control the receptive field allowing the AST encoder to capture multi-view information, we visualize three kinds of mask matrix, two are human-defined (global mask and window mask) and one (structure-induced mask) is from the AST in Figure 3. The global mask (Figure 3a) is a matrix filled with “1”, allowing the node sequence to construct a fully connected graph to capture global information (can be omitted). Each node is able to study from the rest of them. For the structure-induced mask (Figure 3b), each node studies according to the adjacency extracted from the AST and only studies from the node with an edge connection. The window mask is a special mask simulating the sliding window. As shown in Figure 3c, we take a window with size 2 as example. As the window is sliding forward, we are able to capture local information at each window.

### 3.5. Hierarchical Sequence Encoder

A code snippet is some kind of document which consists of several statement sequences. To maintain the document property instead of treating a code snippet as a single sequence, we adopt a hierarchical encoding strategy which captures the code sequence information from token-level to sentence-level.

Given lines of code sub-sequences $S=\{s_{1},s_{2},s_{3},\ldots ,s_{n}\}$, $s_{i}\in R^{m\times dim}$, where *m* denotes the max sequence length between all the sub-sequences and *n* denotes the line number, we are able to capture the hierarchical information using a hierarchical sequence encoder. The procedure is shown in Figure 3.

First, we use the self-attention mechanism to capture the relation between the tokens in each sub-sequence. The first step output $o_{i}$ can be formulated as follows:

(11) $$\begin{array}{c} {REP}_{token}=\left\{SAN\left(s_{1}\right),SAN\left(s_{2}\right),\ldots ,SAN\left(s_{n}\right)\right\} \end{array}$$

where $s_{i}=\left\{{\bar{s}}_{1},{\bar{s}}_{2},\ldots ,{\bar{s}}_{m}\right\}$, ${\bar{s}}_{j}\in R^{dim}$ is the token representation.

Second, to obtain the sentence-level representation, we send the first step output into a long short-term memory network (LSTM), which is able to condense the token-level representation into the sentence-level representation and capture the position information. The final layer of the hidden state in each sub-sequence computation stage is used as the sentence-level representation for each sub-sequence.

(12) $$\begin{array}{c} h_{i}=LSTM\left({Rep^{i}}_{token}\right) \end{array}$$

(13) $$\begin{array}{c} {REP}_{sent}=\left\{h_{1},h_{2},\ldots ,h_{n}\right\} \end{array}$$

where ${Rep^{i}}_{token}\in R^{m\times dim}$, $h_{i}\in R^{1\times dim}$ is the final layer hidden state in the *m*th time step generated by LSTM.

Then, in the same way as step one, we adopt the self-attention mechanism to capture the relation between sub-sequences.

Finally, the second LSTM network has the same effect as the first LSTM network. We use all the final layer hidden states generated in each time step as the code sequence representation.

**Figure 4:**
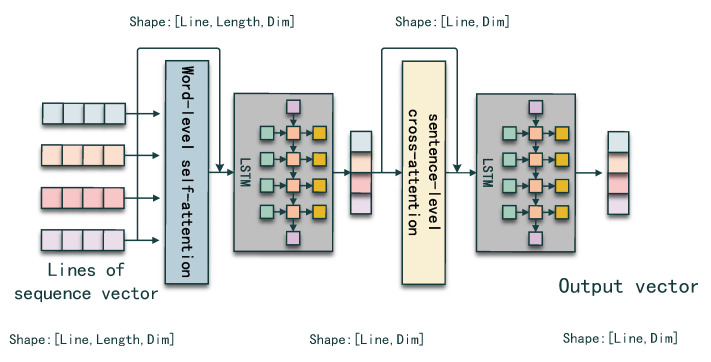
Detail of the hierarchical code sequence encoder.

In Figure 4, we also represent the change of the vector shape in each step to better represent the hierarchical encoding process. At first, the shape of input lines sequence vector is 3D, which has three dimensions ([Line, Length, Dim]), and after the word-level self-attention and LSTM, the vector is compressed into 2D ([Line, Dim]). The final shape remains 2D after the sentence-level self-attention and LSTM modules. Thus, we transform 3D lines of sequence vector into a 2D vector for later combination.

### 3.6. Encoder Output Fusion

In above section, we utilized two encoders for capturing structural information and sequence information. We define the output from the AST encoder as $Encoder_{AST}\left(N,A\right)$ and $Encoder_{S}\left(S\right)$ from the hierarchical sequence encoder.

The SSCS encoder output is obtained by jointing the outputs from the AST encoder and hierarchical code sequence encoder. The computation is as follows:

(14) $$\begin{array}{c} Encoder_{out}=\left[Encoder_{AST}\left(N,A\right):Encoder_{S}\left(S\right)\right] \end{array}$$

where $Encoder_{AST}$ denotes the AST encoder and $Encoder_{S}$ denotes the hierarchical code sequence encoder. *N* refers to the input node vector and *A* is adjacency. *S* refers to the lines of code sequence.

The encoder output will be utilized in the decoding stage for generating the summary.

### 3.7. Bidirectional Decoder

Liu et al. [29] found that the quality of the prefixes of translation hypotheses is much higher than that of the suffixes in machine translation tasks. Furthermore, in order to produce more balanced translations, Liu et al. adopted a simple strategy for joint training the forward decoder and the backward decoder.

Inspired by the previous work of Liu et al., we adopt the same strategy to produce more balanced predictions. We simultaneously generate sequence in the Left-to-Right (L2R) direction and Right-to-Left (R2L) direction. Both directions guide each other by optimizing the shared parameters which are learnable. We define the input vector for the L2R decoder as ${\vec{Y}}_{L2R}=\left\{{\vec{y}}_{1},{\vec{y}}_{2},{\vec{y}}_{3},\ldots ,{\vec{y}}_{n}\right\}$ and ${\overset{\leftarrow }{Y}}_{R2L}=\left\{{\overset{\leftarrow }{y}}_{1},{\overset{\leftarrow }{y}}_{2},{\overset{\leftarrow }{y}}_{3},\ldots ,{\overset{\leftarrow }{y}}_{n}\right\}$ for the R2L decoder. The L2R decoder and R2L decoder can be regarded as two sub-tasks. By optimizing the shared parameters, one task can guide the other one. The details are presented below.

The L2R decoder and R2L decoder share the same Embedding Layer, which converts the one-hot vector of the token into a dense vector:

(15) $$\begin{array}{c} emb\_L2R=Embedder({\vec{Y}}_{L2R}+PE\left({\vec{Y}}_{L2R}\right)) \end{array}$$

(16) $$\begin{array}{c} emb\_R2L=Embedder({\overset{\leftarrow }{Y}}_{R2L}+PE\left({\overset{\leftarrow }{Y}}_{R2L}\right)) \end{array}$$

where Embedder(x)=xW, *W* is a learnable matrix, a converting discrete vector into a continuous vector. PE denotes position encoding operation, used for capturing sequence order.

We define Query,Key,Value in different directions as $\left(\vec{Q},\overset{\leftarrow }{Q}\right),\left(\vec{K},\overset{\leftarrow }{K}\right),\left(\vec{V},\overset{\leftarrow }{V}\right)$. The two directions’ multi-head attention outputs can be computed as below:

(17) $$\begin{array}{c} {\vec{MSA}}_{L2R}=MultiHead\left(\vec{Q},\vec{K},\vec{V}\right) \end{array}$$

(18) $$\begin{array}{c} {\overset{\leftarrow }{MSA}}_{R2L}=MultiHead\left(\overset{\leftarrow }{Q},\overset{\leftarrow }{K},\overset{\leftarrow }{V}\right) \end{array}$$

After the attention computation, the outputs will be sent into a Feed-Forward network, which contains two linear transformations and a ReLU activation function.

(19) $$\begin{array}{c} FFN\left(x\right)=ReLU\left(xW_{1}+b_{1}\right)W_{2}+b_{2} \end{array}$$

By using the Softmax function, we can obtain the probability of the generated token.

In the training stage, the bidirectional guiding decoder generates two directions’ outputs. Our goal is to find the parameter θ which can maximize the likelihood of success. For a training data pair ${\left\{x^{n},{\vec{y}}^{n},{\overset{\leftarrow }{y}}^{n}\right\}}_{t=1}^{N}$, $x^{n}$ is the encoder output, ${\vec{y}}^{n}$ and ${\overset{\leftarrow }{y}}^{n}$ are the decoder inputs. The generation procedure is auto-regressive which means the generation for the next token is based on the previous all generated tokens. We compute the likelihood as follows:

(20) $$\begin{array}{c} P\left(Y|X\right)=\left\{\begin{array}{c} \prod_{t=1}^{n}P\left({\vec{y}}_{t}|{\vec{y}}_{<t-1},X\right)\;if\;L2R\\ \prod_{t=1}^{n}P\left({\overset{\leftarrow }{y}}_{t}|{\overset{\leftarrow }{y}}_{<t-1},,X\right)\;if\;R2L \end{array}\right. \end{array}$$

The final likelihood function is the joint of the likelihood functions of two sub-tasks:

(21) $$\begin{array}{c} \begin{array}{cc} J\left(\theta \right)=\frac{1}{N}\sum_{n=1}^{N}\sum_{j}^{J}\lambda \log p\left({\vec{y}}_{j}|{\vec{y}}_{<j}^{n},{\overset{\leftarrow }{y}}_{<j}^{n},x^{n},\theta \right) & \\ +\left(1-\lambda \right)\log p\left({\overset{\leftarrow }{y}}_{j}|{\overset{\leftarrow }{y}}_{<j}^{n},{\vec{y}}_{<j}^{n},x^{n},\theta \right) \end{array} \end{array}$$

where λ is the weight to balance the guidance of the two sub-tasks. λ decides the training purpose to focus on which direction task more. j denotes the time step and J denotes the whole generating step length.

Different from the training stage, we adopt a beam search strategy for the summary generation. Beam search strategy expands the searching area reserving the best top k token (k denotes the beam size) instead of shrinking the best one as in a greedy search. For each inference step, we generate beam-size candidate sequences and reserve k-best at last. The final sequence is chosen between the two outputs from the L2R decoder and the R2L decoder. Furthermore, we can regard greedy search as a beam search strategy when beam size is 1.

## 4. Experiments and Results

### 4.1. Datasets

Two widely used public datasets are taken into consideration to ensure the performance and generalization ability of SSCS. The Java dataset provided by Hu et al. [6] consists of 87,136 <code, summary> pairs, which is able to test the generation performance on Java language. Furthermore, we also test on a Python dataset provided by Wan et al. [1] containing 92,545 <code, summary> pairs. We follow the procedures of Hu et al. and Wan et al. and divide the datasets into training set, valid set and test set. Table 1 shows the statistics of both datasets including the size for each set and the average length of code and summary in each program language.

### 4.2. Metrics

We evaluate the performance of summarization with BLEU [30], METEOR [31] and ROUGE [32], which are widely used for testing the sentence generation performance in natural language generation (NLG) tasks.

**BLEU** metric is used to calculate the number of n-gram matches between the generated sequence and the reference sequence and calculate the average, n=1,2,...,N. The calculation formula is as follows:

(22) $$\begin{array}{c} P_{n}=\frac{\sum_{gram_{n}\in c}match\left(gram_{n}\right)}{N(gram_{n})} \end{array}$$

(23) $$\begin{array}{c} BLEU=\rho {\left(\prod_{n}P_{n}\right)}^{1/N} \end{array}$$

where $\sum_{gram_{n}\in s}match\left(gram_{n}\right)$ denotes the number of n-grams matches in the generated sequence and the reference sequence *c*. $N(gram_{n})$ denotes the total number of n-grams in the reference sequence, and ρ is the brevity penalty.

**ROUGE** is a recall-based evaluation metric. We use ROUGE-LCS (Longest Common Sequence, ROUGE-L) as our ROUGE evaluation metric in our experiments. ROUGE-LCS takes the longest common sub-sequence between the generated sequence and the reference sequence as the starting point for calculation. The calculation formula is shown below:

(24) $$\begin{array}{c} Recall_{LCS}=\frac{LCS(g,c)}{len(c)} \end{array}$$

(25) $$\begin{array}{c} Precision_{LCS}=\frac{LCS(g,c)}{len(g)} \end{array}$$

(26) $$\begin{array}{c} ROUGE_{LCS}=\frac{\left(1+\beta^{2}\right)Recall_{LCS}Precision_{LCS}}{Recall_{LCS}+\beta^{2}Precision_{LCS}} \end{array}$$

where LCS(g,c) denotes the longest common sub-sequence between the generated sequence *g* and the reference sequence *c*. The size of β determines whether to focus on the Recall rate or Precision rate.

**METEOR** is proposed to solve some inherent defects of BLEU. It uses Word Net to compute specific sequence matches, synonyms, root words and affixes, and paraphrases to make them more relevant to manual judgment.

### 4.3. Hyper Parameters

For the hyper parameter settings, we follow the previous works by Ahmad et al. [8] and Wu et al. [9]. We set embedding size of source code and summary to 512. The layer for the AST encoder is set to 3 and 6 for the summary decoder. We initialize the learning rate as $1\times 10^{-4}$ and use a 4000 step warm-up schedule. The maximum training epoch is set to 200 with an early stop mechanism. The maximum length for code is set to 300 and 100 for the summary. Adam optimizer is used for the optimization of the learning rate. We detail the parameter in Table 2. The experiments are conducted in a server with 4 Nvidia 2080ti GPUs and Ubuntu 18.04 OS (https://www.ubuntu.org.cn/, accessed on 29 January 2023).

### 4.4. Baselines

We compare SSCS with the recent code summarization models and the description of each model is shown as below.

- **RL+HybridSeq** [1] using a critic network with the BLEU score as the reward to conduct the learning of the model.
- **DeepCom** [13] using SBTs traversed from ASTs and code sequence as inputs and using a hybrid attention mechanism to fuse these features.
- **API+CODE** [6] utilizing the API sequence to enhance the representation of code that improved the performance of generating the summary.
- **Dual Learning** [19] utilizing the duality between code generation task and code summarization task and training both tasks simultaneously.
- **Transformer** [8] using a transformer with a copy mechanism and relative positional encoder to generate code summaries.
- **SiT** [9] constructing a multi-view adjacent matrix to represent the relationships between the tokens in the code guiding the self-attention computation.
- **M2TS** [10] constructing a multi-view AST feature at multiple local and global levels and proposing a fusion method to combine sequential information and structural information.
- **SCRIPT** [11] introducing the structural relative positions between nodes of the AST to better capture the structural relative dependencies.
- **CodeScribe** [12] introducing a novel triplet position for AST which is represented by GNNs and using a pointer-generator network to copy tokens from code tokens and tree nodes to summarize.

### 4.5. Results and Analysis

To represent the performance of SSCS, we compare it with eight state-of-the-art baselines. The baseline results are mainly from Ahmad et al. [8] and Wu et al. [9] and the others are from the original papers. The overall result is illustrated in Table 3.

We split the baselines into two groups, one group is the RNN-based baselines, while the others are the transformer-based baselines. Compared with the RNN-based baselines (RL+Hybrid, Deepcom, API+CODE, Dual Model), SSCS is much more superior to them in all evaluation metrics on the Java and Python datasets. Although the recent transformer-based baselines have achieved excellent performance in the code summarization task, SSCS can still perform better than these approaches. Compared with M2TS, SSCS improves the performance of BLEU, METEOR and ROUGE-L by 2.94%, 2.19% and 2.47% on the Java dataset, respectively. Meanwhile, SSCS also exceeds M2TS by 3.64%, 1.56% and 4.43% on the Python dataset. We also conduct a comparison between SSCS and SCRIPT and the result also demonstrates the effectiveness of the SSCS. SSCS improves the performance of BLEU, METEOR and ROUGE-L by 2.89%, 2.64% and 3.65% on the Java dataset and 3.48%, 2.55% and 4.24% on the Python dataset.

Due to the different summary processing strategy between CodeScribe and the other baselines, we compare SSCS with CodeScribe in isolation. CodeScribe replaces all the numerical tokens with a unified symbol ‘<number>’ and removes all the lexical forms (e.g., -s, -es, -ed). The result is shown in Table 4. In the same preprocessing method for the summary, we can see that SSCS still performs better than CodeScribe on the Java and Python datasets. SSCS improves results by 1.36%, 0.96% and 2.91% in BLEU, METEOR and ROUGE-L scores on the Java dataset and 2.20%, 1.22% and 3.39% on the Python dataset.

From the above experiment results, our approach outperforms the current state-of-the-art methods. To better understand the main reasons of the improvement for SSCS, we conduct an ablation study to present the strength of each module we propose in the following section.

### 4.6. Ablation Study

To better understand why our proposed SSCS can achieve such a great performance, the ablation study is necessary for disclosing the main reasons for such improvement.

We first conduct two ablation experiments that remove the important components in SSCS. Thus, the full SSCS model degenerates into the extended Si-Transformer model. By studying the ablation of components, we can directly see the change of scores.

As shown in Table 5, we first remove the bidirectional decoder, which means we only generate the summary from a single direction.Based on this, the performance drops about 2%, 1.2% and 1.4% in BLEU, METEOR and ROUGE-L. Then we remove the sequence encoder and the generation of summary only depends on the structural information. The performance drops about 0.6%, 1% and 1.1% in BLEU, METEOR and ROUGE-L. The ablation study on components illustrates the effectiveness of each component. Furthermore, the bidirectional decoder contributes much in generating better summaries. The hierarchical encoder provides a view at document level, which helps to represent the code comprehensively. IO the left side of Table 5 “w/o” denotes “without” and also means we remove this component in our model. The top row is the full model, so we remove each component step by step.

Moreover, we also conduct experiments of the different fusion strategies for the fusion of the global information, structural information and local information in the AST encoder. The fusion strategies are addition, element-wise dot-product, average and adaptive weight fusion. The result is shown in Table 6, the adaptive weight fusion achieves the best score compared with the other strategies. The element-wise dot-product achieves the lowest score, while performance of the addition strategy is close to the average strategy. It can be obviously seen from the results that the adaptive weight fusion strategy does improve the generation performance.

### 4.7. Validation Performance

To demonstrate the superiority of SSCS, we visualize the validation curve of BLEU and ROUGE scores compared with several baseline. Our approach is based on the transformer architecture; thus, we choose Si-transformer and vanilla transformer as contrasts. The results also contain the generation performance on different generating direction. L2R denotes generating the summary from left-to-right (normal writing order) and R2L denotes the opposite. It is obvious that SSCS achieves a higher score of both languages in the validation set from the first epoch to the last one, which also demonstrates the superiority of SSCS.

### 4.8. Case Study

**Figure 5:**
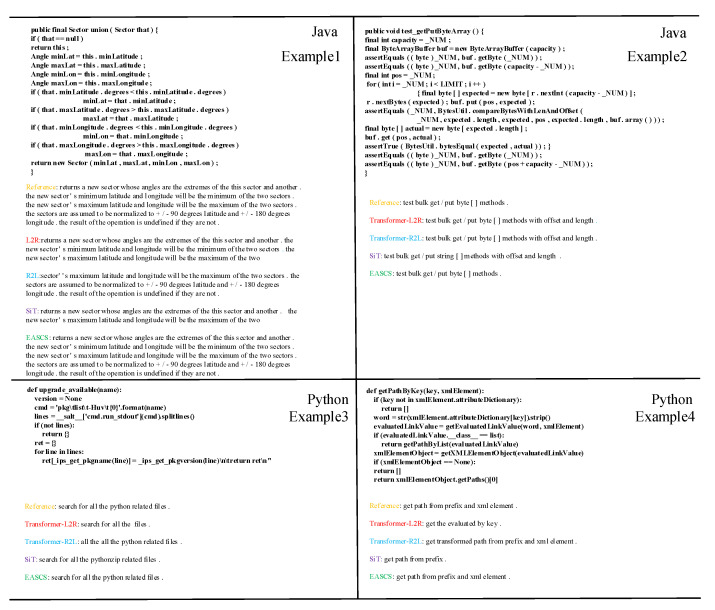
Case example on Java and Python datasets.

Figure 5 shows the qualitative examples of SSCS, SiT, Transformer-L2R (Left-to-Right) and Transformer-R2L (Right-to-Left). It can be observed that Transformer-L2R can generate prefixes well but suffixes poorly, while Transformer-R2L achieves the opposite performance. By utilizing the potential of encoder and decoder, SSCS can generate summaries with balanced prefixes and suffixes. Compared with the SiT, our approach achieves better performance in both Java and Python languages. In general, SSCS is able to generate a more complete and accurate summary. We can clearly see from example 1 (upper left), suffering from exposure bias, L2R and R2L stop generating the summary almost in the middle of the summary, which means the stop symbol ‘<EOS>’ comes up early. However, our proposed approach can overcome the generation hindrance, preventing the stop symbol coming up early, and allowing it to generate the whole summary.

## 5. Conclusions

In this paper, we propose a structure and sequence aligned code summarization model named SSCS which can achieve excellent performance compared with several code summarization baselines. SSCS extends the previous work and utilizes both structural information and sequence information. We design a multi-view mask strategy which enables transformer architecture to capture the AST more comprehensively. Inspired by the works on NMT, we first introduce bidirectional decoding into the code summarization task to release the exposure bias issue, which can generate better summary with both prefixes and suffixes. However, the performance of the automatic code summarization is far from satisfactory; it is still a tough mission to generate high-quality summaries. In future work, we will be devoted to exploring the potential of the encoder and the decoder for the code summarization task. For example, we plan to consider to transform SSCS into a large pre-trained language model. With the great performance achieved by the pre-training strategy, we believe the results will be more satisfying. Furthermore, there is still a long journey for automatically generating a high-quality summary of code.

## 6. Limitations

We have identified the following limitations to our work that may threaten the validity of our work:

- **Baselines Reproduction.** Due to hardware limitations, we cannot reproduce all the baseline methods (e.g., CodeScribe, M2TS). Thus, we process the data using their released tools or use their processed data, and set most of the hyperparameters (e.g., max source length, max target length, max epoch) the same as theirs.
- **Language Type.** We only conduct our experiments on Java and Python, but it is necessary to experiment on other popular languages (e.g., C++, C#, SQL, Rust). We do not know whether the SSCS can achieve the same promotion as on Java and Python.
- **Evaluation Metrics** There are no particular evaluation metrics for code summarization tasks. We follow the previous works on this task evaluating our approach using the metrics in the machine translation task or text summarization task. It is necessary to propose a metric particular for code summarization tasks.

## Figures and Tables

**Table 1:** Statistics of the experimental datasets.

Dataset	Java	Python
Train	69,708	55,538
Validation	8714	18,505
Test	8714	18,502
Avg. tokens in code	120.16	47.98
Avg. tokens in summary	17.7	9.49

**Table 2:** Hyper Parameter Settings.

Param.	Number
max training epoch	200
early stop	20
train batchsize	16
test batchsize	64
max code length	300
max summary length	100
learning rate	0.0001
warm-up step	4000
AST encoder layer	3
decoder layer	6
head	8
embedding size	512
LSTM layer	1
dropout	0.1
optimizer	Adam
beam size	4

**Table 3:** Comparison of our proposed approach with the baselines on Java and Python datasets. Greater values denote better performance.

Model	Java			Python		
	BLEU	METEOR	ROUGE-L	BLEU	METEOR	ROUGE-L
RL+Hybrid2Seq (2018)	38.22	22.75	51.91	19.28	9.75	39.34
DeepCom (2018)	39.75	23.06	52.67	20.78	9.98	37.35
API+CODE (2018)	41.31	23.73	52.25	15.36	8.57	33.65
Dual Model (2019)	42.39	25.77	53.61	21.80	11.14	39.45
Transformer (2020)	44.58	26.43	54.76	32.52	19.77	46.73
Si-Transformer (2021)	45.70	27.55	55.54	33.46	20.28	47.50
M2TS (2022)	46.84	28.93	57.87	33.84	21.83	47.92
SCRIPT (2022)	46.89	28.48	56.69	34.00	20.84	48.15
SSCS	**49.78 **	**31.12**	**60.34**	**37.48**	**23.39**	**52.39**

**Table 4:** Comparison of our proposed approach with CodeScribe. * refers to experiment on the summary processed by Guo et al. [12]. Compared with SSCS, SSCS* takes a cleaner summary which removes all the lexical forms as decoder input.

Model	Java			Python		
	BLEU	METEOR	ROUGE-L	BLEU	METEOR	ROUGE-L
CodeScribe (2022)	49.19	32.27	59.59	35.11	23.48	50.46
SSCS *	50.55	33.23	62.50	37.31	24.70	53.85

**Table 5:** Ablation study on Java and Python Datasets.

Model	Java			Python		
	BLEU	METEOR	ROUGE-L	BLEU	METEOR	ROUGE-L
SSCS	49.78	31.12	60.34	37.48	23.39	52.39
-w/o Bi-decoder	46.70	28.50	57.75	35.24	22.14	50.82
-w/o Hi-encoder	46.10	27.75	57.20	34.64	21.13	49.72

**Table 6:** Performance on different fusion methods on Java and Python Datasets.

Model	Java			Python		
	BLEU	METEOR	ROUGE-L	BLEU	METEOR	ROUGE-L
addition	49.20	30.40	59.70	36.70	22.01	50.46
element-wise dot	47.50	29.10	58.60	34.25	20.10	48.82
average	48.83	30.10	59.42	36.90	22.40	50.82
adaptive weight	49.78	31.12	60.34	37.48	23.39	52.39

## Data Availability

Data sharing is not applicable for this article.

## Abbreviations

The following abbreviations are used in this manuscript:

AST	Abstract Syntax Tree
MSA	Multi-head Self-Attention
SSCS	Structure and Sequence aligned Code Summarization
GNN	Graph Neural Network
